# A Collagen Supplement Improves Skin Hydration, Elasticity, Roughness, and Density: Results of a Randomized, Placebo-Controlled, Blind Study

**DOI:** 10.3390/nu11102494

**Published:** 2019-10-17

**Authors:** Liane Bolke, Gerrit Schlippe, Joachim Gerß, Werner Voss

**Affiliations:** 1 Dermatest GmbH, Engelstraße 37, D-48143 Münster, Germany; dr.bolke@dermatest.de (L.B.); dr.schlippe@dermatest.de (G.S.); 2 Institut für Biometrie und klinische Forschung (IBKF) der Westfälischen Wilhelms-Universität Münster, Schmedding Straße 56, D-48149 Münster, Germany; joachim.gerss@ukmuenster.de

**Keywords:** aging, beauty, bioavailability, collagen peptides, cutometry, corneometry, wrinkles, high coverage [HC] collagen complex

## Abstract

The purpose of this randomized, placebo-controlled, blind study was to investigate the effects of the drinkable nutraceutical ELASTEN^®^ (QUIRIS Healthcare, Gütersloh, Germany) on skin aging and skin health. Drinking ampoules provides a blend of 2.5 g of collagen peptides, acerola fruit extract, vitamin C, zinc, biotin, and a native vitamin E complex. This controlled interventional trial was performed on 72 healthy women aged 35 years or older. They received either the food supplement (*n* = 36) or a placebo (*n* = 36) for twelve weeks. A skin assessment was carried out and based on objective validated methods, including corneometry (skin hydration), cutometry (elasticity), the use of silicon skin replicas with optical 3D phase-shift rapid in-vivo measurements (PRIMOS) (roughness), and skin sonography (density). The verum group was followed for an additional four weeks (without intake of the test product) to evaluate the sustainability of the changes induced by the intake of the test product. The test product significantly improved skin hydration, elasticity, roughness, and density. The differences between the verum group and the placebo group were statistically significant for all test parameters. These positive effects were substantially retained during the follow-up. The measured effects were fully consistent with the subjective assessments of the study participants. The nutraceutical was well tolerated.

## 1. Introduction

Healthy skin provides an active interface between the internal and external environments of the body and enables permanent adaptation and acclimatization of an organism during its lifetime. Many different factors exacerbate the aging process of the skin, including intrinsic ageing, irradiation, consumption of a non-balanced diet, and stress-related deficiencies in micronutrients [1,2,3], leading to an age-dependent collagen loss in the skin.

Collagen, the most abundant component of the extracellular matrix, is the decisive protein that determines skin physiology, by maintaining the skin structure and enabling its numerous functions to take place [4,5,6]. The extracellular matrix retains water and supports a smooth, firm, and strong skin. The structure of collagen is reminiscent of a rope. Three chains wind around each other forming a collagen triple helix. These building blocks combine to form collagen fibrils of enormous strength and tensile force [4,5,6].

Studies have shown that age-dependent reduction in collagen synthesis can be reversed by oral administration of specific bioactive collagen peptides [4,7,8,9,10,11]. These oligopeptides are obtained by enzymatic hydrolysis of natural collagen. After ingestion, they are further metabolized to bioactive di- and tri-peptides in the gastrointestinal tract, which are then released into the blood stream and accumulated in the skin to form the collagen biomatrix [4,7,9]. Typical collagen hydrolysates are composed of peptides of different lengths and, depending on the collagen source, are characterized by a special amino acid composition. The unique (high coverage—[HC]) collagen complex (ELASTEN^®^) tested here contains short chain oligopeptides composed of 5 to 8, 9 to 15, and 16 to 26 amino acids and have a high coverage with amino acids sequences found in human skin collagen proteins.

External and internal factors drive the physiological process of skin aging associated with a decline in collagen formation [12,13]. The collagen content of young and healthy skin has been demonstrated to exceed 75% [4,5,6,12,13]. Collagen fibers are synthesized primarily by the fibroblasts in the deeper layers of the skin. As such, rejuvenation of the biomatrix can be effectively improved only through a supply of sufficient nutrients via the blood stream [2]. Collagen formation is diminished in mature skin and the biomatrix of the skin begins to collapse when the collagen scaffold loses its strength and stability [5,7,9,14]. Factors such as sunlight, smoking, environmental pollution, alcohol abuse, and nutrient deficiency can accelerate this process [12,13]. The elasticity is then diminished, and lines and wrinkles emerge. Due to the loss of collagen, the skin becomes increasingly thinner and drier.

A previous pilot study demonstrated that the oral intake of special bioactive collagen peptides, also tested in the present trial, induce beneficial effects on human skin structure and function [8]. In this former non-controlled pilot trial, which was conducted in 16 healthy women, ELASTEN^®^ was orally applied for 3 months. The study showed significant and sustainable improvements of skin hydration, elasticity, and roughness. The present study was performed to confirm the results obtained in the pilot trial under randomized, placebo-controlled conditions. The peptides provided by the food supplement ELASTEN^®^ are characterized by a high contribution of amino acid sequences from human collagen, and are combined with specific synergistically acting dermonutrients.

## 2. Materials and Methods

### 2.1. Study Design and Ethical Aspects

This clinical study employed a randomized, placebo-controlled, single-blind design. It was conducted according to the applicable principles of good clinical practice (GCP) [15]. The clinical study was registered in the German Clinical Trial Register and at the International Clinical Trials Registry Platform (no. DRKS00015664). The investigation was in full compliance with the principles outlined in the Declaration of Helsinki and with national regulations of Germany. A written informed consent was received from all volunteers. The trial protocol was approved by the Freiburg Ethics Commission International, Freiburg, Germany on 11 September 2017.

### 2.2. Study Participants

A total of 72 female subjects aged 35 years or more with healthy skin of any type participated in this study. The main exclusion criteria were as follows: Major or chronic skin diseases, major internal or chronic diseases, intake of drugs with any impact on skin reactions (e.g., glucocorticoids, antiallergics, and topical immunomodulators), application of preparations and care products containing active substances 7–10 days before participating in the study, heavy allergies or the occurrence of severe adverse events due to cosmetics in the past, sunbathing or solarium visits during the study, known cancer, pregnancy, and lactation.

Participants were instructed to maintain their living habits 9 and to not begin or change any estrogen or progesterone therapies. However, none of the participants were under estrogen or progesterone treatment during the study. Any intake of other products similar to the test product during the study was not allowed.

The recruited subjects were randomized to receive either ELASTEN^®^ (verum group) or a placebo. Randomization was performed with alternating allocation of subjects.

### 2.3. Study Schedule

The study duration was twelve weeks. After obtaining informed consent from the subjects, their suitability for participation in the study was checked. Before the first intake of the study product, after twelve weeks of intake, and after a follow-up period (without intake) of 4 weeks (T16), subjects were dermatologically examined, and tolerability and efficacy data were collected. At the end of the follow-up period, the sustainability of the previously observed effects was evaluated in the test group of subjects who had taken the test product for the twelve-week period.

### 2.4. Test Product and Placebo

The test product is classified as a food supplement. Both the test product and placebo were delivered in identical drinking ampoules. The test product ELASTEN^®^ (QUIRIS Healthcare, Gütersloh, Germany) contained a specially developed blend of 2.5 g collagen peptides, 666 mg acerola fruit extract, 80 mg vitamin C, 3 mg zinc, 2.3 mg vitamin E, and 50 µg biotin. Other ingredients, which were also contained in the placebo, were potassium sorbate, sodium benzoate, carboxymethylcellulose, citric acid, natural aroma, and water. The placebo (QUIRIS Healthcare, Gütersloh, Germany) did not contain any nutrients. The test product and placebo were taken daily before or together with a meal. The specific collagen peptides were produced following GMP (good manufacturing practice) [16] guidelines in an IFS (international featured standards)—and ISO (International Organization for Standardization)—certified plant by water extraction of the endogenous collagen from bovine skin, and subsequent enzymatic hydrolysis and sterilization.

### 2.5. Assessment of Safety and Tolerability

Safety and tolerability of the test material was assessed by three methods: (1) Evaluation of tolerability by dermatological examinations before, during, and after the application phase of the study; (2) monitoring of adverse events during the study through information collected in interviews and questionnaires; and (3) the questionnaire at the end of the study.

### 2.6. Dermatological Examinations

Before, during, and after the study, the skin of subjects was examined according to established clinical and dermatological assessment criteria. Each finding and type of reaction before and after the study was documented and graded on a four-point scale: 0 = no pathological finding, 1 = mild skin reaction, 2 = moderate skin reaction, and 3 = severe skin reaction.

### 2.7. Measurements

All measurements were done under rested conditions of the subjects and under stable physical environmental conditions (room temperature 20 °C and humidity 40%–60%).

#### 2.7.1. Skin Hydration

Hydration of the external layer of the epidermis (stratum corneum) was measured on the forearm capacitively with a Corneometer CM 825 (Courage and Khazaka, Cologne, Germany). For each measurement time, at least three measurements at different locations in the test area on the forearm were performed [17].

#### 2.7.2. Skin Elasticity

For the assessment of skin elasticity, a Cutometer MPA 580 (Courage and Khazaka, Cologne, Germany) was used. The functional principle is based on suction of the skin using a probe with negative pressure, which causes the test area to be drawn into the aperture of the probe [18]. For each measurement, skin elasticity (“R2”) at three different places of the test area (forearm) was assessed and the values were averaged.

#### 2.7.3. Skin Roughness

Skin roughness was measured on the face of the subject, on the basis of silicone imprints (skin replicas). The structure of the skin surface was determined by means of the optical 3D in-vivo measuring method PRIMOS (phase-shift rapid in-vivo measurement of skin; PRIMOS Compact, GFMesstechnik GmbH, Teltow, Germany). The “arithmetic average roughness” and the “maximum roughness” were used as common skin surface descriptors. A replica was prepared for each measurement in order to determine skin roughness Rz, which is defined as arithmetic mean of the individual depths of five contiguous individual measuring sections of the (digitally) filtered profile of equal lengths. The technology reproducibly determines skin roughness [19].

#### 2.7.4. Skin Density

Sonographic measurements were used to determine skin density [14]. The SkinScanner DUB^®^ Simple (tpm^®^ taberna pro medicum, Lüneburg, Germany) enables the visualization of structures up to a maximal depth of 1 cm. Measurements were performed on the thigh.

### 2.8. Questionnaire

After 12 and 16 weeks, participants filled out questionnaires regarding their subjective assessments of different parameters concerning the characteristics and performance of the product such as efficacy, odor, taste, consistency, and skin appearance.

### 2.9. Statistical Analysis

Statistical analyses were performed according to the principles of ICH (International Conference on Harmonization) guideline E9 “Statistical Principles for Clinical Trials” [20] using SAS software (Version 9.4 for Windows, SAS Institute Inc., Cary, NC, USA).

The objective skin parameters—hydration, elasticity, roughness, and density—were evaluated by descriptive analyses at T0 (day 0), T12 (day 84), and T16 (day 112). Efficacy was determined by relative changes of these parameters, which were determined by the following differences of the means: T12–T0, T16–T0, and T16–T12. The location and scale statistics of all the parameters were calculated, including the arithmetical mean, standard deviation, minimum, and maximum. From the calculated values of skewness and kurtosis it was concluded that all the parameters could be regarded as (approximately) normally distributed.

The results are presented graphically by means of box-and-whisker plots. The box ranges from the 25^th^ percentile up to the 75^th^ percentile. Whiskers are drawn from the ends of the boxes to the largest and smallest values that did not represent outliers. Outliers are defined as being values more than 1.5 times the interquartile range away from the box. Outliers are represented by symbols beyond the whiskers.

Inferential statistical analyses were performed using Student’s *t* test [21]. Intra-individual mean changes of skin parameters (comparing T12 versus T0, T16 versus T0, and T16 versus T12) were evaluated using paired *t* tests. The mean outcomes in the verum and placebo groups were compared using the *t* test for independent samples. The primary outcomes and test hypotheses of the trial were based on the comparison of the verum and placebo groups with respect to the relative change (T12–T0) of: (i) Skin hydration, (ii) skin elasticity, (iii) skin roughness, and (iv) skin density. The multiple significance level across the four primary tests was set to 5% (two-sided). In order to adjust for multiple testing, the Bonferroni method [22] was applied. The primary results provide confirmatory statistical evidence. Beyond the primary statistical analyses, all other statistical analyses are considered exploratory. The *p*-values are regarded noticeable (“significant”) for *p* ≤ 0.05.

## 3. Results

### 3.1. CONSORT (Consolidated Standards of Reporting Trials) Flow Diagram of the Controlled Interventional Trial

**Figure 1:**
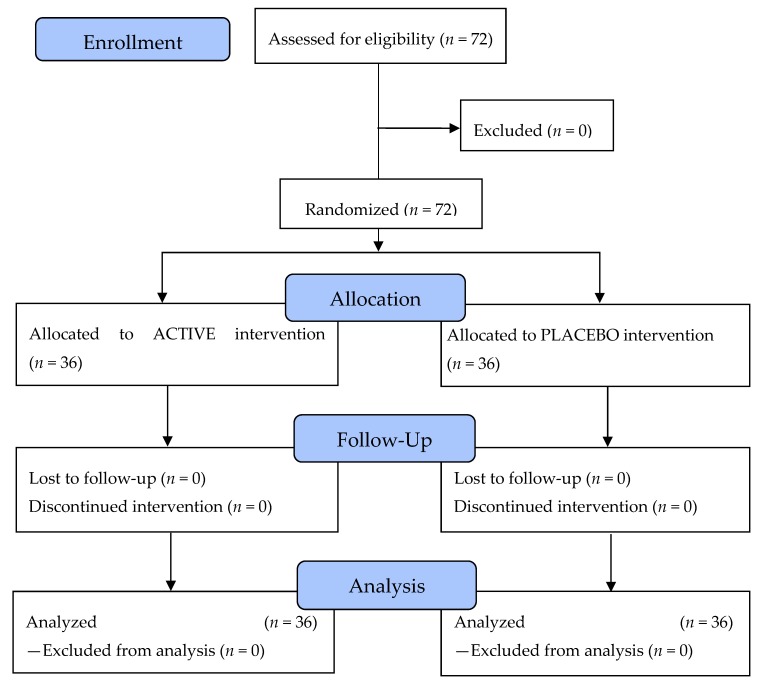
Recruitment of the eligible subjects with the intervention protocol and assessment.

Seventy-two subjects aged 35 to 73 years were statistically analyzed. The mean age in the verum group (*n* = 36) was 50.6 ± 11.2 years and in the placebo group (*n* = 36), 52.4 ± 8.3 years. No subjects had to be excluded during screening and throughout the study. No protocol violations occurred. All subjects assessed for eligibility could be analyzed (*n* = 72). Hence, the intention-to-treat (ITT) population and the per-protocol (PP) population were identical. The safety population (SP) also included all 72 enrolled subjects. The flow of subjects through the controlled interventional trial is depicted in a CONSORT conform diagram [23] (Figure 1).

The clinical study was conducted as a two-arm clinical interventional trial of the test product against placebo for twelve weeks with a follow-up period of four weeks without intake in the subjects that received the test product. At the end of the follow-up period, the sustainability of the previously observed effects was demonstrated in the test group that was no longer treated with the test product. All enrolled subjects were examined as intended before first intake of the test product or placebo (T0), after twelve weeks of intake of test product or placebo (T12), and after a follow-up period without intake of four weeks in subjects that had previously taken the test product (T16).

### 3.2. Tolerability

The products were well tolerated. Dermatological examinations revealed no pathological skin reactions, irritancy reactions, or allergic reactions. The tolerability of the test product/placebo was rated by subjects as follows: “very good” = 66%/56%, “good” = 33%/33%, “neither nor” = 0%/8%, “bad” = 0%/3%, and “very bad” = 0%/0%. In the placebo group, one subject reported nausea and one heartburn. Apart from these two cases, participants did not report any adverse events throughout the study and follow-up period.

### 3.3. Skin Hydration

**Figure 2:**
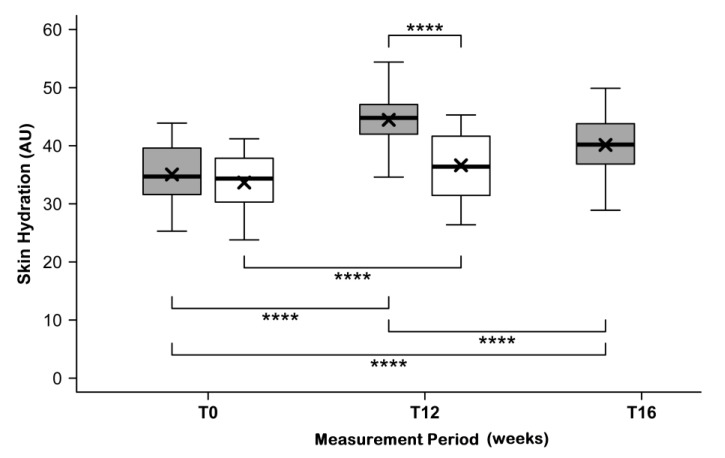
Skin hydration before (T0) and after (T12) intake of study products, as well as after follow-up (at T16) for the test product (grey) and placebo (white) groups. The boxplot shows the mean (×), median ( 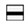), and max–min whiskers (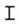 ). AU indicates arbitrary units, where absolute levels and changes are significantly different for the test product and placebo with *p* ≤ 0.0001 (****).

Figure 2 shows the descriptive analysis of skin hydration before intake of study products (at T0), after twelve weeks of intake (at T12), and at the end of the follow-up period (at T16). No significant differences were found for the mean initial skin hydration at T0 for the verum group and the placebo group (35.0 ± 4.8 AU vs. 33.7 ± 5.1 AU, *p* = 0.2405). At T12, mean skin hydration was significantly increased, by 28.0% ± 11.5% (44.5 ± 4.4 AU vs. 36.6 ± 5.7 AU, *p* < 0.0001), in the verum group. On the other hand, the increase in the placebo group was limited to 9.0% ± 6.6%. The difference between the groups at T12, as well as the difference between the relative changes in skin hydration of both groups proves to be highly significant (*p* < 0.0004) in favor of the test product.

The final measurements at T16 show that such effects persist for some time. After four weeks without further taking of the test product, skin hydration was still significantly increased in comparison to the initial state (40.1 ± 4.9 AU).

### 3.4. Skin Elasticity

**Figure 3:**
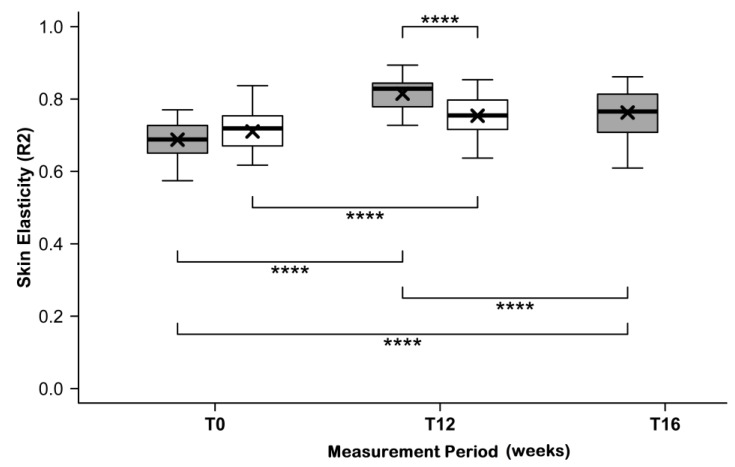
Skin elasticity before (T0) and after (T12) intake of study products, and after follow-up (at T16) for the test product (grey) and placebo (white) groups. The boxplot shows the mean (×), median (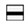 ), and max–min whiskers ( 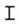). The absolute levels and changes are significantly different for the test product and placebo groups, with *p* ≤ 0.0001 (****).

At T0, skin elasticity was similar in the verum group (R2 = 0.69 ± 0.05) and placebo group (R2 = 0.71 ± 0.06; *p* = 0.3240). After intake of the test product and placebo, the elasticity (R2 values) significantly increased in both groups (Figure 3). At T12, skin elasticity was increased in the verum group by 0.81 ± 0.04 AU and in the placebo group by 0.75 ± 0.06 AU. This difference proved to be highly statistically significant (*p* < 0.0004).

Furthermore, skin elasticity in the test group was only slightly reduced at the end of the follow-up period (R2 = 0.76 ± 0.07), corresponding to a mean decline of −6.38% ± 7.01%, however it did not drop to its initial level.

### 3.5. Skin Roughness

**Figure 4:**
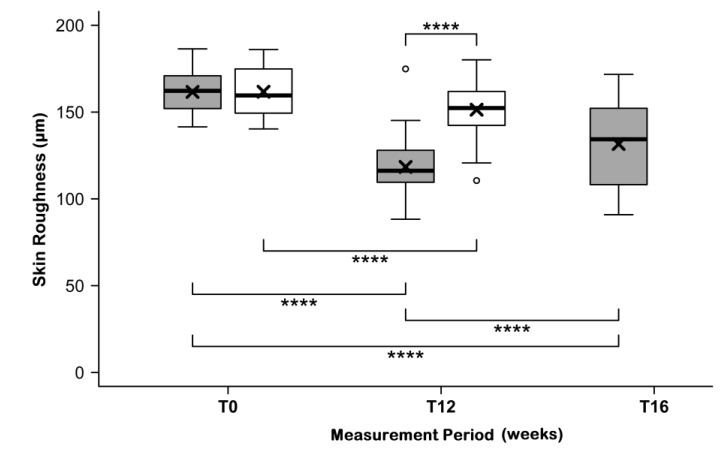
Skin roughness (wrinkle depth determined from the phase-shift rapid in-vivo measurement of skin (PRIMOS) skin replica) before (T0) and after (T12) intake of study products and after follow-up (FU) with test product (grey) and placebo (white), boxplot showing the mean (×), median ( 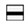), and max-min whiskers ( 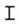), absolute levels and changes are significantly different for the test product and placebo at *p* < 0.0001 (****). ^O^ represent outlier.

In line with improvements in skin hydration and elasticity, skin roughness decreased during intake of the test product. As shown in Figure 4, the depth of wrinkles measured by PRIMOS replicas was reduced in the verum group after twelve weeks of intake. Starting from similar initial mean values of 161.6 ± 11.4 µm (test product) and 161.7 ± 13.0 µm (placebo; *p* = 0.9702), the wrinkle depth decreased to 118 ± 16.4 µm in the verum group and to 151.4 ± 15.9 µm in the placebo group. The relative difference between T0 and T12 was four times higher in the verum group (−26.8% ± 8.1%) than in the placebo group (−6.4% ± 5.8%; *p* < 0.0004). The maximum improvement of skin roughness was 41%.

After the follow-up period, the depth of wrinkles increased between T12 and T16 from 118 ± 16.4 µm to 131.6 ± 21.9 µm, demonstrating a persistent, highly significant difference of −18.9% ± 9.9% in skin roughness in relation to the initial state (Figure 4; *p* < 0.0001). 

### 3.6. Skin Density

**Figure 5:**
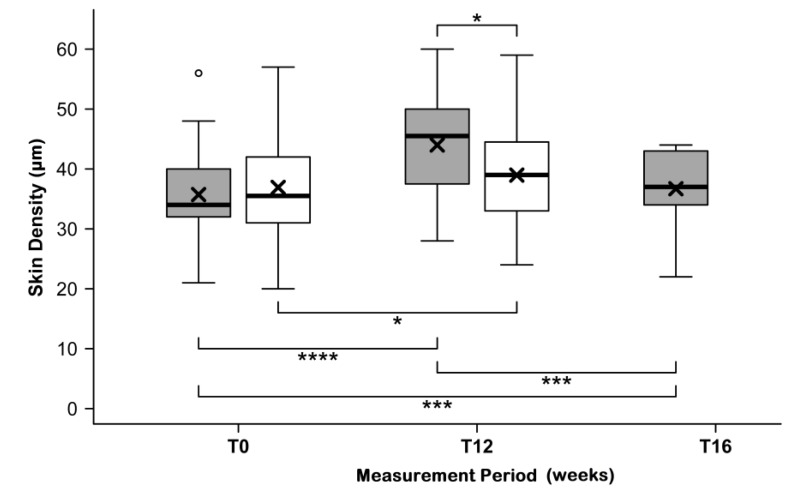
Skin density (sonographically measured) before (T0) and after (T12) intake of study products, and after follow-up (at T16) for the test product (grey) and placebo (white) groups. The boxplot shows the mean (×), median ( 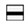), max–min whiskers (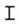 ), and outliers (o). Absolute levels and changes are significantly different for the test product and placebo with *p* ≤ 0.0001 (****), *p* ≤ 0.001 (***), and *p* ≤ 0.05 (*). ^O^ represent outlier.

Both test product and placebo induced an improvement in the skin density, sonographically measurable as the thickness of the epidermis. Starting from similar initial values (35.7 ± 7.2 µm for the test product and 36.9 ± 8.6 µm for placebo), measurements revealed a highly significant increase in the thickness of the epidermis and the corresponding skin density in the verum group by 24.8% ± 16.8% (to 44.0 ± 7.6 µm, *p* < 0.0001; Figure 5). Furthermore, a significant improvement was observed in the placebo group of 6.8% ± 14.8% (to 39.0 ± 8.7 µm, *p* < 0.0109). The difference between the relative changes of both groups proved to be highly significant (*p* < 0.0004) in favor of the test product.

The effect of the test product within twelve weeks did not disappear during the four weeks of the follow-up period. After discontinuing the application, the mean skin density decreased to 36.7 ± 6.9 µm at T16. This value was still a highly significantly increase compared to the initial value at T0 (*p* = 0.0008).

### 3.7. Persistence of Skin Improvements

**Figure 6:**
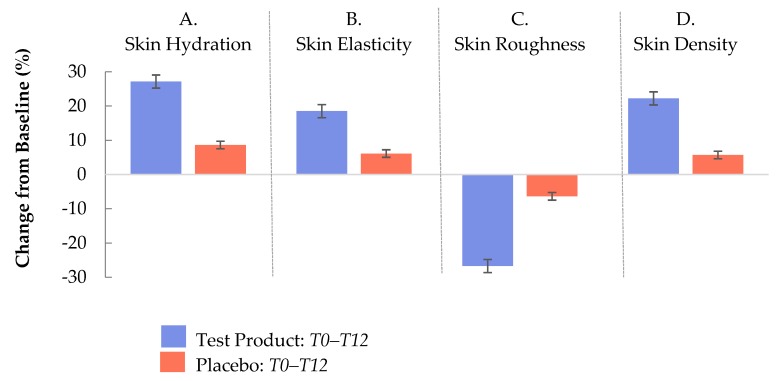
Percentage change of skin parameters between the baseline and the end of the twelve week interventional period (T0–T12) in the test group (blue bars) and in the placebo group (red bars). Error bars indicate the standard errors of the mean. Changes are significant at *p* < 0.0001 (all parameters except skin density at T16 with a significance level of *p* < 0.0008).

**Figure 7:**
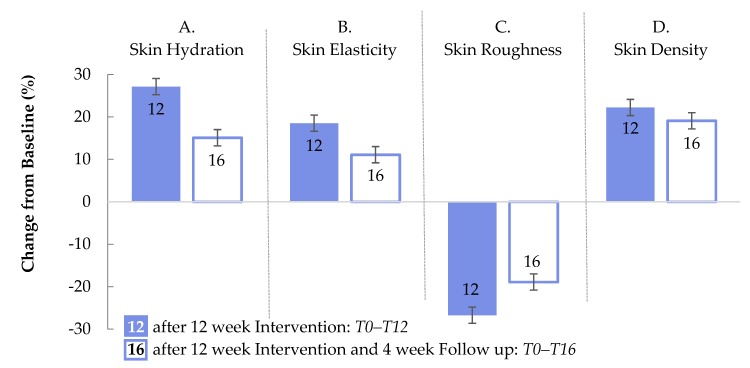
Percentage changes of skin parameters in the test group between baseline and the end of the twelve week interventional period (T0–T12, solid blue bars) and between baseline and the end of the four week follow-up period (T0–T16, open white bars) for skin hydration (A), skin elasticity (B), skin roughness (C), and skin density (D) in the test group. The error bars indicate standard errors of the mean. Changes are significant at *p* < 0.0001.

The efficacy and sustainability of the effects of the test product are summarized in Figure 6 and Figure 7. In Figure 6, the differences between the changes (T0–T12) in skin hydration (A), skin elasticity (B), skin roughness (C), and skin density (D) of the test product and placebo are illustrated. The relative differences between T0 and T12 were significantly higher in the verum group than in the placebo group *(p* < 0.0004).

After discontinuation of administration of the test product, the effect on skin hydration was reduced by −45.8% during the follow up compared to T12. A better persistence was demonstrated for the improvement of skin roughness. This parameter decreased by only 30.6% compared to T12. Skin elasticity and skin density were reduced by 38.5% and 31.3%, respectively (Figure 7). For all skin parameters tested in this study, the percentage improvements in the verum group at the end of the follow-up period (T16, Figure 7) were much more pronounced than the improvements in the placebo group at T12 (Figure 6).

### 3.8. Subjective Rating

In the beginning, about 64% of all participants characterized their skin as more or less dry, approximately 22% as normal, and about 6% as fatty (8% did not provide such information). All prompted effects were more positively assessed by the test group as compared to the placebo group. Up to three quarters of subjects in the test group agreed with the positive statements. As shown in Table 1, the differences of the test group compared to the placebo group were most apparent regarding scaly skin (47% more agreements), skin elasticity (23%), skin appearance (29%), and skin dryness (13%). After discontinuation of taking the study products, experienced effects largely persisted for at least four weeks in both study groups.

## 4. Discussion

Topically applied skin care products such as creams, lotions, and sera often fail to reach the deeper layers of the skin in order to causally and lastingly influence the skin aging processes. The aim to reach the dermis, the most important skin layer for the restoration of collagen synthesis, has been achieved by developing highly bioavailable and thus bioactive short chain nutritional collagen peptides [4,5,10,24]. This was also confirmed by the present trial, particularly by the improvement of the density and elasticity of the skin. It was clearly shown that the effects of the test product were not restricted to the epidermis.

A systematic review of the dermatological applications of oral collagen supplementation has recently demonstrated that collagen supplements can increase skin hydration, elasticity, and dermal collagen density. Eleven studies with a total of 805 patients suggest that administration of collagen peptides can positively impact various skin conditions and skin aging [9]. Collagen supplementation was safe with no reported adverse effects. Asserin et al. demonstrated in a randomized controlled trial that the intake of 10 g of collagen hydrolysate over at least 56 days leads to an increase in skin moisture and collagen density compared to placebo [25]. Collagen dipeptides that were taken over 56 days in a study by Inoue et al. also resulted in significantly more improvement in skin moisture, elasticity, wrinkles, and roughness for the subjects that received 10 mg per day in comparison to study participants who received 0.5 mg per day or placebo [26].

Compared to placebo, a significant reduction in eye wrinkle volume along with an increase of procollagen type I and elastin was reported by Proksch et al. after the intake of 2.5 g collagen per day over 56 days [27,28]. In addition, it was shown by Schunck et al. the intake of 2.5 g collagen peptides over a period of 180 days led to a statistically significant decrease in the degree of cellulite and a reduced skin waviness on thighs [29].

The supplement tested here provides a drinkable blend of collagen peptides and acerola fruit extract with endogenous antioxidants, such as vitamin C, zinc, biotin, and a native vitamin E complex. Studies suggest that an age-related reduction in collagen synthesis can be addressed by the administration of oral collagen peptides together with other specific skin nutrients [7,9,11]. 

A clinical pilot study has previously demonstrated significant effects of the test product on skin hydration, elasticity, and roughness after three months of application of the supplement in 16 women aged 45–60 years [8].

The present study was conducted as a clinical interventional study on 72 healthy women treated either with the food supplement (*n* = 36) or a placebo (*n* = 36) for three months to confirm and extend these observations. It was demonstrated that drinkable collagen peptides together with other dermonutrients could induce long lasting visible improvements in the skin’s appearance. However, it should also be considered that collagen peptides, depending on the source material and manufacturing process, could differ with respect to molecular size and amino acid composition. 

One reason for the significant improvements of skin parameters shown in the present study might be the high similarity between the collagen peptides provided by the bovine [HC] collagen complex and those of human collagen. Hydrolysis of the bovine collagen yields specific bioactive short chain peptides that are characterized by a high coverage of their amino acid profile with the amino acid sequence of human collagen I. The amino acid sequence coverage of the bovine collagen peptides used in this study was determined by liquid chromatography–mass spectrometry (nanoLC/MS/MS hardware [24]: Aquity^®^ UPCL M-Class HSS T3, Waters, MA, USA) using Mascot software (Matrix Science, London, UK). This analysis revealed a coverage of 31% for collagen type I alpha 1 chains, 18% for collagen type I alpha 2 chains, and 13% for collagen type III alpha 1 chains. These values were compared to 4% to 20%, 7% to 16%, and 6% to 11% ranges, respectively, for other collagen peptides, obtained from pigs, chickens, or marine sources (see Reference [30] for a review).

Moreover, the amino acid composition of the peptides is reflected by a high content of specific amino acids that are abundant building blocks of human collagen, such as hydroxyproline, proline, glycine, glutamic acid, alanine, and arginine [7,31,32]. During digestion, the oligopeptides are further metabolized to bioactive di- and tri-peptides in the gastrointestinal tract and are subsequently released into the blood stream [11,33,34,35,36,37,38]. The concentration and type of collagen, as well as the content of other skin-relevant nutrients can differ in mono and combination products. Therefore, it is crucial to underline the importance of product-specific trials.

In the follow-up phase, the verum group was observed one month after cessation of the treatment to evaluate the sustainability of the changes induced by the application. The results show that all the effects were substantially retained during the follow-up period. Throughout the study, there were no adverse effects, neither for the placebo nor for the test product, which has been on the market for many years as a food supplement.

The placebo effects shown here were attributed to the unconscious lifestyle changes of the subjects during the study and conclusively demonstrated the necessity of a placebo control to reliably assess the effects of a test product. The differences between the verum group and the placebo group were highly significant for all of the test parameters after three months of treatment. For all of the skin parameters tested in this study, the percentage improvement in the verum group was still higher at the end of the follow-up period, after four months, than at the beginning of the application.

The findings were relevant in terms of skin physiology and decisive for demonstrating the validity of this specific approach. Moreover, the effects were not only fully confirmed in objective test methods for assessing skin hydration, elasticity, roughness, and density, but also in the subjective assessment of the subjects. Therefore, the oral application of dermonutrients allows for a long-lasting regeneration of the skin that is clearly visible and cosmetically relevant.

## 5. Conclusions

This randomized, placebo-controlled clinical trial confirmed that skin aging could be addressed using nutrients that are able to restore skin hydration, elasticity, and density. Objective dermatological measurements, such as cutometry and corneometry, have proven that oral collagen peptides together with other dermonutrients significantly improve skin hydration, elasticity, roughness, and density after three months of intake. These tests thus verify the results obtained in previous trials. Moreover, and in line with the objective measurements, the study participants, in their subjective assessments, concluded that their skin appearance had significantly improved. 

Finally, the collagen supplement did not cause any side effects and proved to be safe and well tolerated during the entire period of application and thereafter.

Since the collagen peptides and the skin nutrients were taken orally, the effects reached the deeper layers of the skin and sustainably improved skin physiology and appearance. In conclusion, the tested dermonutrient allowed for a long-lasting and cosmetically relevant regeneration of the skin. Ongoing studies are exploring the decisive nutritional mechanisms involved in improving skin physiology and appearance.

## Figures and Tables

**Table 1:** Self-perception rating, agreement to statements concerning test product effects reported as a percentage in relation to placebo effects.

After 12 Weeks of Taking the Product	Δ (%) Test Group Relative to Placebo Group
my skin is less dry.	+13.0
my skin is less scaly.	+47.4
the elasticity of my skin is improved.	+22.7
my skin appearance is improved.	+28.6
my skin appears more wrinkle-free.	+10.5
external skin care products can be used less frequently.	+27.3

Δ = Delta, difference in percent after 12 weeks of intake.
